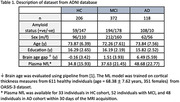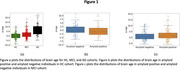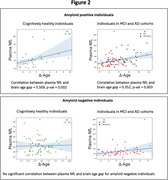# Brain age correlates with plasma NfL in amyloid positive individuals

**DOI:** 10.1002/alz.089232

**Published:** 2025-01-09

**Authors:** Saurabh Sihag, Alejandro Ribeiro

**Affiliations:** ^1^ University of Pennsylvania, Philadelphia, PA USA

## Abstract

**Background:**

Brain age prediction using machine learning (ML) models provides novel opportunities to characterize neurobiology of accelerated aging. Brain age gap, i.e., the gap between the predicted age and the chronological age for an individual, is a meaningful metric to assess accelerated or premature aging. Plasma neurofilament light (NfL) is a well‐recognized biomarker that can reflect the severity of structural atrophy and the loss of white matter integrity in Alzheimer's disease. Here, we leveraged an anatomically interpretable ML pipeline for brain age prediction (see ref. [1]) to investigate the correlations between brain age and plasma neurofilament light chain (NfL) in older adults in the ADNI cohort.

**Method:**

Brain age was derived from the cortical thickness measures (Desikan‐Killiany atlas, available online as part of the ADNI database) for 206 healthy individuals (HC), 372 individuals with MCI diagnosis, and 118 individuals with dementia diagnosis (AD). Further, contributions from individual brain regions to the predicted brain age were characterized for each individual. The amyloid status was available for all individuals and plasma NfL (within 30 days of the MRI scan acquisition) was available for a subset of individuals (Table 1). The ML model used to derive brain age had been trained on the healthy individuals from the OASIS‐3 dataset. We assessed the associations between brain age and plasma NfL for HC and the combined MCI and AD cohorts.

**Result:**

Brain age was significantly elevated in AD cohort with respect to HC cohort (Table 1, Figure 1). No significant differences were found between the brain age gap for amyloid positive and amyloid negative individuals (Figure 1). For HC cohort, brain age was positively correlated with plasma NfL in amyloid positive individuals (with brain age contributions from entorhinal region exhibiting the most significant positive correlation (ρ=0.72, p=2.45e‐6) with plasma NfL), but not amyloid negative individuals (Figure 2). In the combined MCI and AD cohort, brain age was correlated with plasma NfL in only amyloid positive individuals.

**Conclusion:**

Our results suggest that amyloid status may mediate the association between plasma NfL and brain age in older individuals.

[1] Sihag et al., Explainable Brain Age Prediction using coVariance Neural Networks, NeurIPS, 2023.